# Effectiveness of T-Cell Replete Haploidentical Hematopoietic Stem Cell Transplantation for Refractory/Relapsed B Cell Acute Lymphoblastic Leukemia in Children and Adolescents

**DOI:** 10.3389/fped.2021.743294

**Published:** 2021-10-13

**Authors:** Hideki Sano, Kazuhiro Mochizuki, Shogo Kobayashi, Yoshihiro Ohara, Nobuhisa Takahashi, Shingo Kudo, Tomoko Waragai, Kazuhiko Ikeda, Hitoshi Ohto, Atsushi Kikuta

**Affiliations:** ^1^Department of Pediatric Oncology, Fukushima Medical University Hospital, Fukushima, Japan; ^2^Department of Blood Transfusion and Transplantation Immunology, Fukushima Medical University School of Medicine, Fukushima, Japan

**Keywords:** acute lymphoblastic leukemia, haploidentical hematopoietic stem cell transplantation, graft-versus-host disease (GVHD), graft versus leukaemia effect, anti-thymocyte globulin (ATG)

## Abstract

**Background:** The prognosis of refractory/relapsed B-cell precursor acute lymphoblastic leukemia (BCP-ALL) remains dismal owing to acquired resistance to chemotherapeutic agents. This study aimed to evaluate the efficacy of T-cell replete HLA haploidentical hematopoietic stem cell transplantation (TCR-haplo-HSCT) for pediatric refractory/relapsed BCP-ALL (RR-BCP-ALL).

**Methods:** Nineteen pediatric patients with RR-BCP-ALL underwent TCR-haplo-HSCT between 2010 and 2019 at the Fukushima Medical University Hospital. The disease status at TCR-haplo-HSCT included complete remission (CR) in eight patients and non-CR with active disease in 11 patients. Total body irradiation-based, busulfan-based, and reduced-intensity conditioning regimens were employed in 11, 6, and 2 patients, respectively. Low-dose anti-thymocyte globulin (thymoglobulin, 2.5 mg/kg) was used in all patients. Graft-vs.-host disease (GVHD) prophylaxis was administered with tacrolimus, methotrexate, and prednisolone.

**Results:** All patients received peripheral blood stem cells as the stem cell source. The HLA disparities in graft vs. host directions were 2/8 in one, 3/8 in five, and 4/8 in 13 patients. Among 18 patients who achieved primary engraftment, acute GVHD occurred in all 18 evaluable patients (grade II, 9; grade III, 8; grade IV, 1), and chronic GVHD was observed in 10 out of 15 evaluable patients. Three patients died because of transplant-related mortality. The 3-year overall survival (OS) and leukemia-free survival rates were 57.4 and 42.1%, respectively. Compared to patients older than 10 years in age (*N* = 10), those younger than 10 years in age (*N* = 9) showed an excellent OS rate (3-year OS rate: patients < 10 years old, 100%; patients > 10 years old, 20% [95% confidence interval, 3.1–47.5]; *p* = 0.002).

**Conclusions:** We suggest that TCR haplo-HSCT with low-dose ATG conditioning has the potential to improve the transplantation outcomes in patients with RR-BCP.

## Introduction

In the treatment of pediatric B-cell precursor acute lymphoblastic leukemia (BCP-ALL), patients who fail to achieve complete remission (CR) after relapse, who experience a primary induction failure, or who relapse after hematopoietic stem cell transplantation (HSCT) have extremely poor prognosis (1). Allogeneic HSCT is accepted as the curative treatment option for relapsed or refractory BCP-ALL (RR-BCP-ALL). However, the prognosis of high-risk patients exhibiting early or very early relapse or those positive for minimal residual disease (MRD) is unsatisfactory even if hematological remission is achieved at the time of HSCT (2, 3). In addition, it is extremely difficult to treat HSCT in patients who fail to achieve remission induction after relapse. In recent years, the treatment of RR-BCP-ALL has dramatically changed owing to development of novel therapeutic agents such as blinatumomab, inotuzumab, and CD19 chimeric antigen receptor T (CAR-T) cells (4–6). However, even with the introduction of such novel therapies, some patients fail to achieve remission or have residual MRD; therefore, further development of treatment methods is required.

While HLA haploidentical HSCT (haplo-HSCT) increases the probability of finding a family donor and greatly facilitates donor selection, it is known to be associated with the development of severe graft-vs.-host disease (GVHD) and early transplant-related mortality (TRM) owing to graft rejection (7, 8). Szydlo et al. (9) reported that TRM was significantly higher after haploidentical related or one antigen-mismatched unrelated donor transplants than after HLA-identical sibling transplants. However, unmanipulated haplo-HSCT using post-transplant cyclophosphamide (PTCY) or anti-thymocyte globulin (ATG) has been developed in the last 2 decades. Consequently, TRM due to early post-transplant complications has decreased and the safety of haplo-HSCT has improved. However, the graft-vs.-leukemia (GVL) effect is attenuated in haplo-HSCT using PTCY or high-dose ATG because of suppression of the T-cell-mediated allogeneic immune response. We consider T-cell-replete haplo-HSCT (TCR-haplo-HSCT) using low-dose ATG to be a form of T-cell therapy that has a high degree of efficacy in hematological malignancies based on the allogeneic immune reaction. We had previously reported the outcomes of TCR-haplo-HSCT with low-dose ATG for relapsed or refractory acute pediatric leukemia in 2018 (10), but the outcomes of TCR-haplo-HSCT for BCP-ALL could not be clarified at that time. Thus, in this study, we aimed to evaluate the efficacy of TCR-haplo-HSCT with low-dose ATG for pediatric RR-BCP-ALL by adding new patients to the former cohort.

## Materials and Methods

### Patients

Nineteen patients with RR-BCP-ALL who received unmanipulated haplo-HSCT from a family donor between 2009 and 2019 at Fukushima Medical University Hospital were retrospectively analyzed (Table 1). Thirteen of the 19 patients in this study were the same as those in the cohort of our 2018 report (10) and were included here with updated survival information. After adding six new patients, the safety and efficacy of TCR-haplo-HSCT were evaluated for patients with RR-BCP-ALL. We excluded Philadelphia chromosome (Ph)-positive and infant ALL patients from the present analysis. The institutional review board approved the study protocol, and written informed consent was obtained from the patients or their guardians and family donors. Follow-up for all patients was continued through May 2021.

**Table 1:** Patient, donor, and graft characteristics.

**Pt. No**.	**Age at HSCT (years)/ Sex**	**BFM risk at first relapse**	**Cytogenetics**	**Disease status at HSCT**	**Donor**	**HLA disparity in GVH**	**Stem cell source**	**TNC (10^**8**^/kg)**	**CD34+ cells (10^**6**^/kg)**	**CD3+ cells (10^**8**^/kg)**
1	1.7/F	Very early BM (S4)	*KMT2A-AFF1*	CR2	Father	3/8	PB	32.1	14.2	10.0
2	2.9/M	Early BM (S3)	Hyperdiploid	Active disease	Mother	4/8	PB	18.4	5.7	5.0
3	4.1/M	Very early combined (S4)	*TCF3-HLF*	EM active disease	Father	4/8	PB	21.5	17.8	10.0
4	6.1/F	Relapse after HSCT	*KMT2A-MLLT3*	CR2 after 1^st^ HSCT	Father	4/8	PB	16.5	6.3	5.9
5	6.1/F	Very early BM (S4)		CR2	Father	3/8	PB	32.4	10.3	9.1
6	6.7/M	Very early BM (S4)	*TCF3-PBX1*	CR2	Mother	3/8	PB	13.6	15.1	5.1
7	6.8/M	Early BM (S3)	Hyperdiploid	CR2	Father	4/8	PB	8.5	8.3	1.2
8	8.8/F	Late BM (S2)	*ETV6-RUNX1*	EM active disease	Father	4/8	PB	17.9	12.5	5.6
9	9.7/M	Early BM (S3)	Hyperdiploid	Active disease after 1^st^ HSCT	Father	3/8	PB	21.2	20.2	5.8
10	10.0/F	Early BM (S3)		Active disease	Father	2/8	PB	11.3	11.2	4.4
11	11.5/M	Late BM (S2)	*IKZF1* deletion	Active disease after 1^st^ HSCT	Father	4/8	PB	15.3	10.2	4.7
12	11.9/F	Early BM (S3)		CR2	Father	4/8	PB	15.6	11.5	5.3
13	12.0/M	Late BM (S2)	Hyperdiploid	Active disease after 1^st^ HSCT	Father	4/8	PB	10.8	13.2	4.4
14	12.3/M	Very early BM (S4)		Active disease	Mother	4/8	PB	13.9	6.0	2.5
15	12.5/F	Very early BM (S4)	Hypodiploid	Active disease	Father	4/8	PB	15.2	10.5	3.6
16	12.5/M	PIF		Active disease with PIF	Mother	4/8	PB+BM	13.7	3.7	2.1
17	12.8/M	Early BM (S3)		CR3 after 1^st^ HSCT	Father	4/8	PB	19.8	12.9	8.4
18	13.9/M	Late BM (S2)	Hypodiploid	Active disease after 1^st^ HSCT	Father	4/8 KIR+	PB	14.0	7.4	3.0
19	16.9/M	Very early BM (S4)	*MEF2D-BCL9*	CRi	Father	3/8	PB	10.1	6.3	4.3

*Pt., patient; BM, bone marrow; CR, complete remission; CR2, second complete remission; CR3, third complete remission; CRi, Complete remission with incomplete hematologic recovery; F, female; HSCT, hematopoietic stem cell transplantation; KIR+, killer cell immunoglobulin-like receptor (KIR) ligand mismatch positive; M, male; PB, peripheral blood; TNC, total nucleated cell count*.

*Berlin-Frankfurt-Münster (BFM) risk stratification for first relapse: very early (<18 months from diagnosis, S4), early (18 months from diagnosis but <6 months after treatment completion, S3), and late (≥6 months after treatment completion, S2)*.

### Donor Sources

Donors included fathers (*n* = 15) and mothers (*n* = 4) of the patients. HLA-A, HLA-B, HLA-C, and HLA-DRB1 typing was performed using PCR-Luminex (Luminex Corporation, Austin, TX, USA), based on reverse sequence-specific oligonucleotide (PCR-rSSO) technology (Genosearch HLA, Medical & Biological Laboratories Co., Ltd., Nagoya, Japan). HLA-disparities in both graft-vs.-host and host-vs.-graft directions included two loci mismatches in one patient, three loci mismatches in five patients, and four loci mismatches in 13 patients. Peripheral blood stem cells (PBSCs) were collected with apheresis using COBE Spectra or Spectra Optia (Terumo BCT, Tokyo, Japan) and bone marrow (BM) cells were collected from related donors using standard mobilization protocols. The target amount of CD34-positive and CD3-positive cells was at least 5.0 × 10^6^/kg and 5.0–10.0 × 10^8^/kg, respectively. Eighteen patients received granulocyte colony stimulating factor (G-CSF)-mobilized PBSCs solely as a stem-cell source, but one patient received BM cells in addition to PBSCs because of low CD34 cell count among the PBSCs.

### Conditioning Regimen and GVHD Prophylaxis

Myeloablative conditioning was administered to 17 patients {total body irradiation (TBI) -based for 11 and busulfan (BU)-based for 6 patients}, whereas reduced-intensity conditioning was administered to 2 patients who had organ dysfunction or active infection. For the patients of first transplantation, we employed the regimen consisting of 12 Gy TBI, 1,800 mg/m^2^/day (>30 kg) or 60 mg/kg/day (<30 kg) intravenous etoposide (VP-16) and 120 mg/kg/day intravenous cyclophosphamide (CY). Patients who had TBI based regimen during the first transplantation received the conditioning regimen consisting of intravenous BU 3.2–4.0 mg/kg/day for 4 days, intravenous fludarabine 30 mg/m^2^/day for 5 days and intravenous melphalan 70 mg/m^2^/day for 2 days. No lung-shielding was performed on patients who underwent TBI. Our GVHD prophylaxis method has been previously reported (11). To prevent GvHD, all patients received ATG (thymoglobulin, 1.25 mg/kg/day; Sanofi, Paris, France) intravenously for 2 consecutive days, from days−2 to−1. GVHD prophylaxis comprised a combination of tacrolimus, methotrexate, and prednisolone for all patients. Tacrolimus was started on day−1 and was continuously administered intravenously. The concentration of tacrolimus in the peripheral blood was adjusted between 7 and 15 mg/ml. 3 or 4 weeks after transplantation, tacrolimus administration was changed to the oral route with the trough level targeted at 5–10 ng/ml. MTX (10 mg/m^2^) was administered intravenously on day +1, and doses of 7 mg/m^2^ were administered on days +3 and +6 after transplantation. Prednisolone (PSL) was initiated on day 0 with an initial dose of 1 mg/kg/day (Patient No. 1 received 2 mg/kg). When there was no sign of acute GVHD, from day +29, the PSL dose was tapered every 2 weeks and discontinued within 6 months after transplantation. Acute GVHD was graded according to the standard criteria (12). The diagnosis and grading of chronic GVHD followed National Institutes of Health (NIH) criteria (13).

### Supportive Care

All patients received prophylaxis with trimethoprim–sulfamethoxazole against *Pneumocystis jirovecii* infection. They received broad-spectrum antibiotics, fluconazole, and acyclovir for bacterial, fungal, and herpes virus infections, respectively. Immunoglobulin (0.2 g/kg/dose, i.v.) was infused weekly until day +100, and then biweekly until 6 months after HSCT. Granulocyte-colony stimulating factor (G-CSF) (5 mg/kg/day) was started on day +1 following stem cell infusion. Cytomegalovirus (CMV) treatment with ganciclovir was initiated when CMV antigenemia was detected in routine weekly examination.

### Analysis of Mismatched HLA Loss

Mismatched HLA loss was detected using the PCR-rSSO-Luminex method. The measured data were read using a dedicated software (DNASIS^®^ Call HLA typing software, Hitachi Software Engineering Co., Ltd. Tokyo, Japan).

### Statistical Analysis

Overall survival (OS) was defined as the time from TCR-haplo-SCT to death from any cause. Relapse-free survival (RFS) was defined as the time from TCR-haplo-SCT to leukemia relapse or any cause of death. Moderate-severe chronic GVHD/ relapse free survival (CGRFS) was defined as the duration from transplantation until death, relapse, development of moderate or severe chronic GVHD that required systemic treatment, and the patients without any of these events at the time of the final follow-up were censored. OS and RFS were calculated using the Kaplan–Meier method (14). The median value of the distribution was used for the age and CD3 cutoff used in the univariate analysis. The cumulative incidences of relapse, TRM and acute GVHD were estimated by analyzing competing risks using Gray's method (15). The Fisher's exact test was used for categorical variables and non-parametric test (Mann-Whitney test) was used for continuous variables. Statistical significance was set at p < 0.05. All statistical analyses were performed using EZR (Saitama Medical Center, Jichi Medical University, Saitama, Japan) (16), which is a graphical user interface for R (The R Foundation for Statistical Computing, Vienna, Austria).

## Results

### Patient, Donor, and Graft Characteristics

Patient, donor, and graft characteristics are presented in Table 1. The median age of patients at the time of TCR-haplo-HSCT was 10.0 (range: 1.7–16.9) years old. Twelve patients (63%) were male, and seven (37%) were female. Among 19 patients with RR-BCP-ALL, eighteen patients relapsed, and one experienced primary induction failure (PIF). The Berlin-Frankfurt-Münster (BFM) risk classification at first relapse was very early BM or combined (S4) in 7 patients, early BM (S3) in 6 patients, late BM (S2) in 4 patients, and post-HSCT relapse in 1 patient. Thirteen patients who relapsed exhibited cytogenetic aberrations. Of the thirteen, four exhibited hyperdiploidy, two exhibited hypodiploidy, and one each exhibited *KMT2A-MLLT3, TCF3-PBX1, IKZF1 deletion, KMT2A-AFF1, ETV6-RUNX1, and MEF2D-BCL9*. At the time of TCR-haplo-HSCT, eight patients achieved CR, while 11 patients had a non-remission status; of the latter 11 patients, nine patients had BM involvement and two had only extramedullary involvement.

### Infused Cell Number and Engraftment

PBSCs were used as the stem-cell source in all patients and were collected from the patients' family (three from mothers and fifteen from fathers). The median number of CD34-positive hematopoietic cells was 10.2 × 10^6^ (range: 3.7–20.2) cells/kg. We used unmanipulated PBSCs to prevent attenuation of the GVL effect and intentionally administered CD3-positive cells; the median dose of CD3 positive cells was 5.0 × 10^8^ (range: 1.2–10.0) cells/kg. Eighteen patients (95%) achieved primary neutrophil engraftment after transplantation. The median time of neutrophil recovery (>500/μL) was 13 (range: 10–16) days and that of platelet recovery (>2 × 10^4^/μL) was 23 (range: 7–123) days. One patient who exhibited primary induction failure (Patient No. 8) experienced donor type primary graft failure; because primary graft failure was not based on donor specific antibody (DSA), he underwent a second BMT from the same donor and achieved neutrophil engraftment 16 days after the second BMT. In this study, there were no other patients with positive DSA.

### HLA Disparities and GVHD

**Figure 1:**
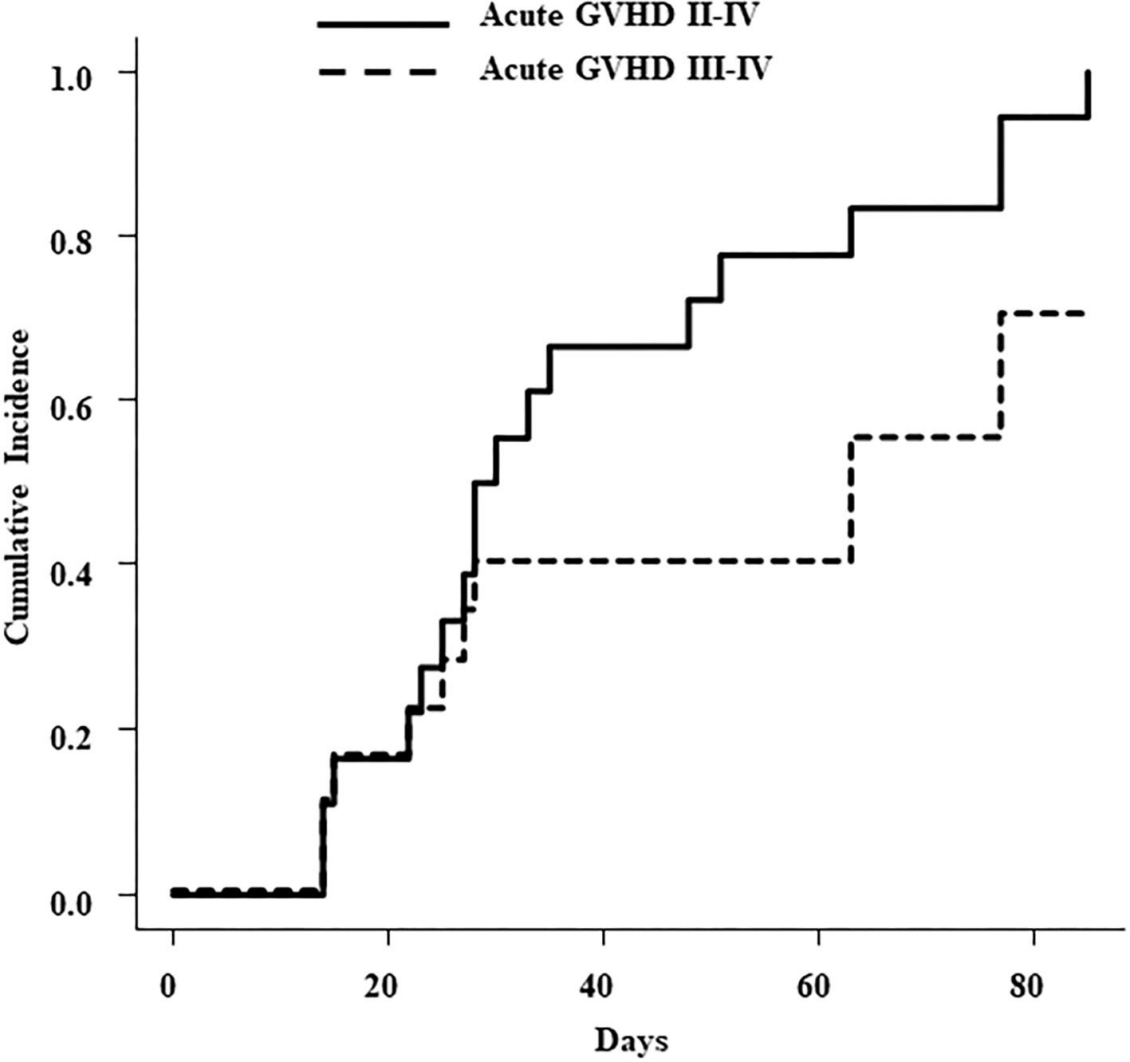
Cumulative incidence of acute GVHD with grade II to IV (solid line) and grade III to IV (dotted line).

HLA disparities in the graft-vs.-host direction were 2/8 in one, 3/8 in five, and 4/8 in 13 patients. In Patient No. 18, killer cell immunoglobulin-like receptor (KIR) ligand mismatch was found in HLA-C between the donor and the recipient. Acute GVHD was observed in all 18 evaluable patients; there were nine patients with grade II, eight with grade III, and one with grade IV GVHD. The cumulative incidence of acute GVHD (grade II-IV) was 100% and 70.1% in that of grade III-IV GVHD (Figure 1). Chronic GVHD was also observed in 10 (67%) of 15 evaluable patients. With reference to chronic GVHD, five patients developed mild, one developed moderate, and four had severe forms with bronchiolitis obliterans (BO). Three of the four patients who developed BO were under 10 years old, and BU was used in two of the three patients. One of these patients (Patient No.1) avoided TBI because of younger age, and the other (Patient No.4) had a BU based regimen because TBI had been used in the first HSCT.

### Complications Within 100 Days After TCR-Haplo-HSCT

Fifteen patients had infectious complications within 100 days of TCR-haplo-HSCT, including CMV antigenemia, Epstein-Barr virus (EBV) reactivation, human herpes virus 6 reactivation, hemolytic cystitis due to BK virus, varicella-zoster virus reactivation, sepsis, and *Aspergillus* infection. Patient No. 14 died because of pulmonary hemorrhage due to invasive aspergillosis on day 23 after TCR-haplo-HSCT, and Patient No. 18 died because of sepsis caused by severe acute GVHD on day 87 after TCR-haplo-HSCT (Table 2). Engraftment syndrome (ES) was observed in 5 patients; two of these five patients relapsed, but both survived with CR after the second TCR-haplo-HSCT.

**Table 2:** T-cell replete haploidentical stem cell transplantation and clinical outcomes.

**PatientNo**.	**Conditioning regimen**	**Engraftment (days)**		**aGVHD grade and stage (skin, liver, gut)**	**cGVHD**	**Complication at <100 days after TCR-haplo-HSCT**	**Post-HSCT relapse (day+)**	**Outcomes**
		**Neutrophil**	**Platelet**					
1	Bu4+Flu+Mel+ATG	10	14	III (2,0,2)	Severe (lung)	ES, EBV	No	Alive with CR
2	TBI(12)+VP16+CY +ATG	15	28	II (3,0,0)	Mild	ES, sepsis (*S. hemolyticus*), HHV6, EBV, PRES	405 (combined)	Alive with CR after 2^nd^ HSCT
3	Bu4+Clo+Flu+ATG	14	123	III (2,0,3)	Mild	ES, CMV, EBV	No	Alive with CR
4	Bu4+Flu+Mel+ATG	11	23	III (2,0,3)	Severe (lung)	BKV-HC, EBV, VZV	No	Alive with CR
5	TBI(12)+VP16+CY +ATG	11	18	II (3,0,1)	Mild	ES	323 (combined)	Alive with CR after 2^nd^ HSCT
6	TBI(12)+VP16+CY +ATG	14	19	III (1,0,3)	Severe (lung)	ES, CMV, aspergillus, atelectasis,	No	Alive with CR
7	TBI(12)+VP16+CY +ATG	11	93	II (3,0,0)	None	CMV	670 (BM)	DOD on day+1986
8	TBI(12)+VP16+CY +ATG	12	19	II (3,0,0)	Mild	None	No	Alive with CR
9	Bu4+Flu+Mel+ATG	15	28	III (3,2,0)	None	BKV-HC, sepsis (*K. pneumoniae*)	No	Alive with CR
10	TBI(12)+VP16+CY +ATG	15	NE	III (3,2,2)	NE	*Candida* sepsis, pancreatitis, aspergillus	67 (BM)	DOD on day+133
11	Bu4+Flu+ATG	13	74	II (3,0,0)	Mild	CMV, EBV, BKV-HC, VZV	105 (BM)	DOD on day+213
12	TBI(12)+VP16+CY +ATG	12	22	II (3,0,0)	None	CMV	No	Alive with CR
13	TBI(12)+VP16+CY +ATG	16	34	II (3,0,0)	Moderate	None	No	Died on day+439 from EB-LPD
14	TBI(12)+VP16+CY +ATG	13	NE	NE	NE	Lung bleeding due to invasive aspergillosis	No	Died on day+23 from aspergillosis
15	Flu+Mel+ATG	11	21	IV (0,4,0)	None	EBV, CMV, BKV-HC, cellulitis, pneumoniae	176 (BM)	Died on day+276 from GF in 2^nd^ HSCT
16	TBI(12)+VP16+CY +ATG	NE	NE	II (3,0,0)	NE	VZV	No	Alive with CR
17	Bu2+Flu+Mel+ATG	10	7	III (2,0,3)	Severe (lung)	EBV, CMV, pancreatitis	115 (CNS)	Died on day+934 from pneumoniae
18	Bu4+Mel+AraC+ATG	13	32	II (3,0,0)	None	None	117 (BM)	DOD on day+549
19	TBI(12)+VP16+CY +ATG	13	NE	III (3,0,3)	NE	Sepsis (*S. oralis*), pancreatitis, HC	No	Died on day+87 from sepsis due to GVHD

*aGVHD, acute graft vs. host disease; ATG, anti-thymocyte globulin; BKV, BK virus; BM, bone marrow; Bu2, busulfan for 2 days; Bu4, busulfan for 4 days; cGVHD, chronic GVHD; Clo, clofarabine; CMV, cytomegalovirus; CNS, central nervous system; CR, complete remission; CY, cyclophosphamide; DOD, died of disease; ES, engraftment syndrome; EBV, Epstein-Barr virus; Flu, fludarabine; GF, graft failure; GVHD, graft vs. host disease; HC, hemorrhagic cystitis; LPD, lymphoproliferative disease; Mel, melphalan; NE, not evaluable; PRES, posterior reversible encephalopathy syndrome; TBI, total body irradiation; TBI (12), TBI 12 Gy; TCR-haplo-HSCT, T-cell replete haploidentical hematopoietic stem cell transplantation; VP16, etoposide; VZV, varicella-zoster virus*.

### Transplantation-Related Mortality, Relapse, and Outcome

**Figure 2:**
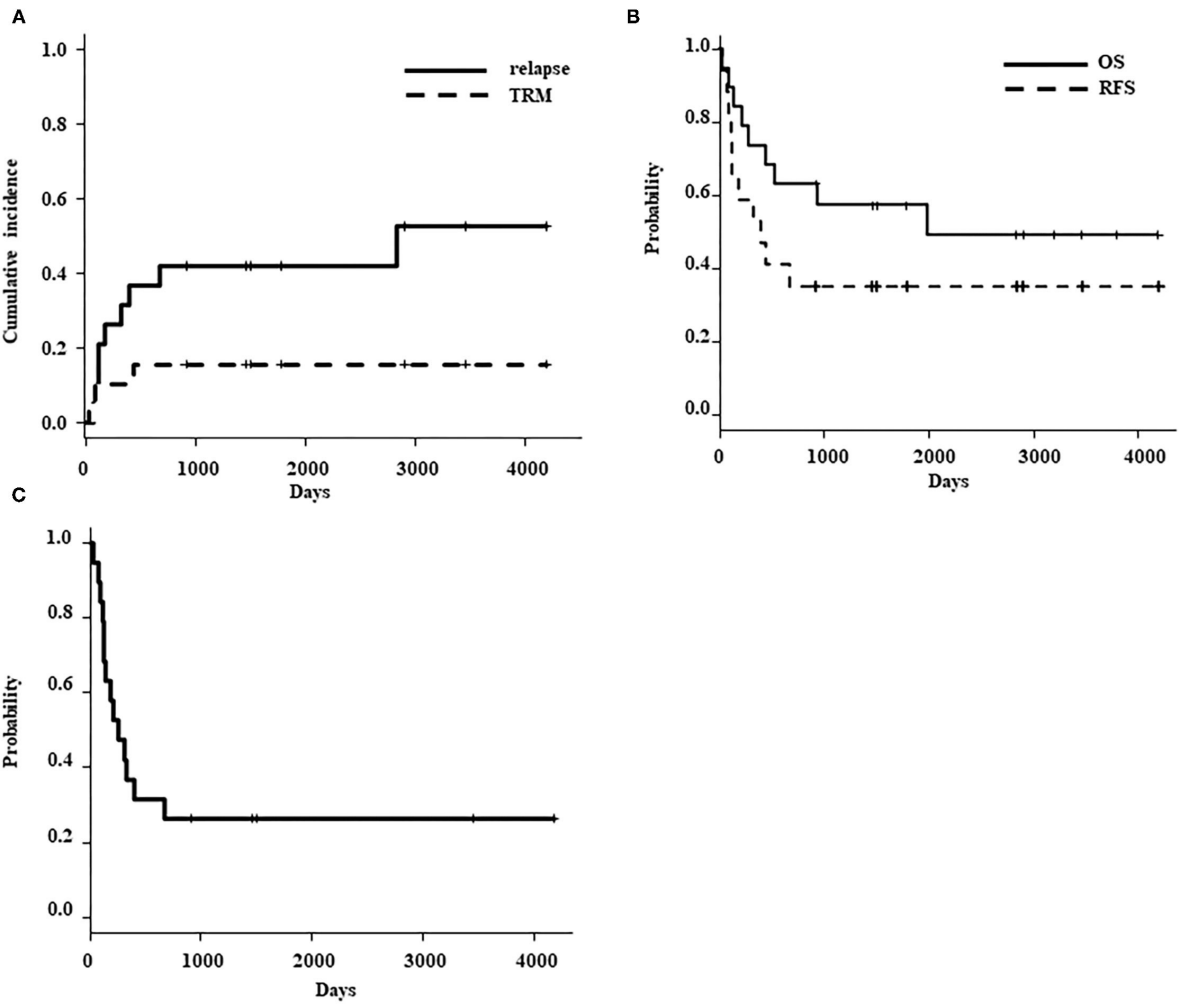
**(A)** Cumulative incidence of relapse (solid line) and transplant-related mortality (dotted line). **(B)** Probability of overall survival (solid line) and relapse-free survival (dotted line). **(C)** Probability of moderate-severe chronic GVHD/ relapse free survival (CGRFS).

The cumulative incidence of relapse at 3 years was 42.1% (95%CI, 19.5–63.3), and the TRM rate was 15.8% (95%CI, 3.7–35.7) (Figure 2A). Five patients died owing to reasons other than leukemia progression; two of these patients died from complications that occurred after the post-HSCT relapse. Therefore, we determined that three patients died from TRM. The causes of TRM were as follows: pulmonary hemorrhage due to invasive aspergillosis, pneumonia due to severe chronic lung GVHD, and sepsis due to severe acute GVHD. Characteristics of patients who relapsed after TCR-haplo-HSCT have been summarized in Table 3. Eight patients experienced leukemia relapse; two (Patient No. 2 and 5) of the four patients who received the second TCR-haplo-HSCT survived, and all four patients who did not receive a re-transplantation died due to disease progression. Of the five relapsed patients for whom HLA analysis of blasts was available, three had mismatched HLA loss after TCR-haplo-HSCT and all three died; the two patients without mismatched HLA loss were rescued by the second TCR-haplo-HSCT after relapse. Interestingly, Patient No. 4 received TCR-haplo-SCT from his father but relapsed with loss of the maternal HLA haplotype; he then underwent haplo-SCT from his mother and relapsed with loss of the paternal HLA haplotype (17).

**Table 3:** Summary of relapsed patients after T-cell replete haploidentical stem cell transplantation.

**PatientNo**.	**Age at TCR-haplo-HSCT (years)**	**Relapse site**	**Mismatched HLA haplotype loss**	**2^**nd**^ TCR-haplo-HSCT (donor)**	**Outcome**
2	2.9	Combined	(-)	YES (uncle)	Alive with CR
5	6.1	Combined	(-)	YES (uncle)	Alive with CR
7	6.8	BM	NE	NO	DOD day+1986
10	10.0	BM	(+)	NO	DOD day+133
11	11.5	BM	NE	NO	DOD day+213
15	12.5	BM	(+)	YES (mother)	Died from graft failure at 2^nd^ HSCT
17	12.8	CNS	NE	NO	Died from pulmonary complication
18	13.9	BM	(+)	YES (mother)	DOD day+549

*BM, bone marrow; CNS, central nervous system; NE, not evaluable; CR, complete remission; DOD, died of disease; TCR-haplo-HSCT, T cell replete haploidentical hematopoietic stem cell transplantation*.

### Survival

**Figure 3:**
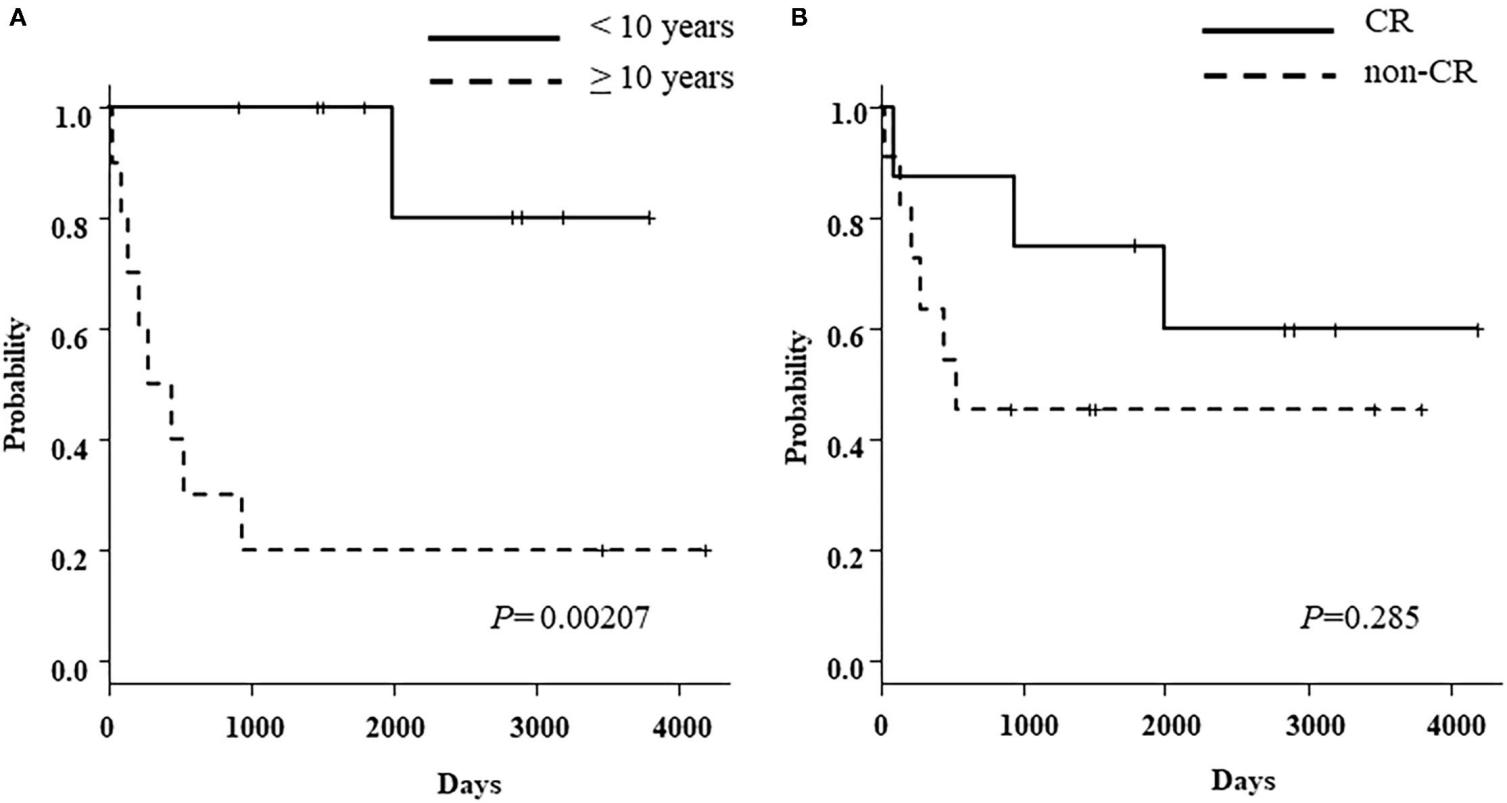
**(A)** Probability of overall survival according to the age at TCR-haplo-HSCT [<10 years (solid line) vs. more than 10 years (dotted line)] **(B)** Probability of overall survival according to the disease status at TCR-haplo-HSCT [complete remission (solid line) vs. non-complete remission (dotted line)].

As of the last follow-up date, ten of the 19 patients were still alive after a median follow-up of 2,866 (range: 913–4,190) days. The probability of OS and RFS at 3 years was 57.4% (95%CI, 32.5–76.0) and 42.1% (95%CI, 20.4–62.5), respectively (Figure 2B). The probability of 3 years CGRFS was 26.3% (95%CI, 9.6–46.8) (Figure 2C). Patients younger than 10 years (*N* = 9) exhibited an excellent overall survival rate compared to patients older than 10 years (*N* = 10) [3-year OS: patients <10 years old, 100%; patients >10 years old, 20% (95%CI, 3.1–47.5); *p* = 0.002] (Figure 3A). However, the disease status at the time of TCR-haplo-HSCT was not associated with the patient's prognosis [3-year OS: CR, 75% (95%CI, 31.5–93.1); NCR, 45.5% (95%CI, 16.7–70.7); *p* = 0.285] (Figure 3B). We also examined whether there was a difference in patient characteristics between the patients <10 and those >10 years old (Table 4). There was no significant difference between the two groups except for the CD3 dose, which may be confounded by the very small number of cases. Moreover, this result needed to be interpreted carefully because the number of patients in the study cohort was too small to perform multivariate analysis. All three patients who died of TRM were over 10 years in age, and all five patients over 10 years in age who relapsed after TCR-haplo-SCT died; furthermore, all three patients who relapsed with mismatched HLA loss were also over 10 years in age (Table 3). Our case series included 13 high-risk patients with a BFM risk classification of very early (S4) and early (S3) for the first relapse; seven of these patients are still alive. Additionally, high infused CD3 dose was also associated with better survival [3 years OS: >5.0 × 10^8^/kg (*N* = 10), 88.9% (95%CI, 43.3–98.4); <5.0 × 10^8^/kg (*N* = 9), 22.2% (95%CI, 3.4–51.3); *p* = 0.0003]. The CD3 infusion dose itself was correlated with age; therefore, caution should be exercised when considering CD3 infusion dose as an independent prognostic factor. It is important to note that infusing more CD3 does not increase mortality due to complications.

**Table 4:** Comparison of patient characteristics by age.

**Variables**	**<10 years (*n* = 9)**	**≥10 years (*n* = 10)**	***P*-value**
**Sex**			
Male	5	3	0.65
Female	4	7	
**Donor**			
Father	7	8	1.00
Mother	2	2	
**Disease status at TCR-haplo-SCT**			
CR	5	8	0.37
Active disease	4	2	
**HLA disparity**			
2/8	0	1	0.21
3/8	4	1	
4/8	5	8	
**CD3 dose**			
MedianRange	5.8 × 10^8^/kg (1.2–10.0)	4.35 × 10^8^/kg(2.1–8.4)	0.025
**CD34 dose**			
MedianRange	12.5 × 10^6^/kg (5.7–20.2)	10.35 × 10^6^/kg(3.7–13.2)	0.22

*TCR-haplo-SCT, T cell replete haploidentical hematopoietic stem cell transplantation*.

## Discussion

Relapse after first-line chemotherapy occurs in 15−20% of pediatric patients with BCP-ALL (18), and survival rates are much lower in cases of BM relapse with a shorter duration from diagnosis than in late and/or extramedullary relapse (19). In the BFM risk classification, very-early relapse is defined as that occurring <18 months from diagnosis, early relapse as that occurring more than 18 months from diagnosis but <6 months from treatment discontinuation, and late relapse as that occurring more than 6 months from treatment discontinuation (20). The 5-year OS rates of early BM (S3) and very-early BM or combined (S4) relapse are very low at 30 and 25%, respectively (21). Patients with high-risk relapses have poor survival rates even after allogeneic HSCT. The pre-HSCT assessment of MRD in patients with high-risk relapse (S3/S4 or third CR) showed that patients with MRD of <10^−4^ leukemic cells had significantly better survival (53% probability of event-free survival [pEFS]) than patients with MRD of over 10^−4^ leukemic cells (30% probability of pEFS) (22). Historically, patients who could not achieve CR were not considered for HSCT because of the lack of prognostic improvement after treatment. Furthermore, TCR-haplo-HSCT has been shown to be associated with a higher risk of early TRM, owing to complications in severe GVHD arising from allogeneic immune reactions and graft rejection. Therefore, HSCT from HLA-matched donors has traditionally been recommended. In contrast, allogeneic immune reactions induce a strong GVL effect; thus, TCR-haplo-HSCT may be considered as a way to induce GVL in patients with relapsed/refractory leukemia.

The outcome of pediatric BCP-ALL has been gradually improved by the stratification of treatment based on the analysis of MRD and the introduction of new drugs. However, it is difficult to improve the prognosis of patients who have relapsed after HSCT, of patients with early or very early relapse who have high chemo-resistant disease, and of patients with failure to induce remission after treatment with conventional HSCT. In order to treat such cases, we have developed a unique method to maximize the GVL effect of TCR-haplo-HSCT as a cell-mediated immunotherapy for refractory/relapsed acute leukemia including non-CR cases by reducing the amount of ATG, optimizing GVHD prophylaxis, and adding a high-dose of T-cell infusion. We retrospectively analyzed a case series of relapsed and refractory leukemia treated with TCR-haplo-HSCT and reported it in 2018 (10). In the present study, we added six new cases to the 13 previously reported cases and evaluated the efficacy of TCR-haplo-HSCT only in RR-BCP-ALL.

Although the analysis was based on a small number of patients (*N* = 19), the 3-year OS and RFS were 57.4 and 42.1%, respectively, despite including 11 extremely high-risk patients with active disease as non-CR cases. A noteworthy aspect of our study is that the 3-year OS of patients with non-CR (*N* = 11) was 45.5% (95%CI, 16.7–70.7). There were three non-CR patients under 10 years of age, all of whom are still alive. In our previous report on relapsed/refractory acute leukemia in children, the age at transplantation (<10 years vs. ≥10 years) was considered an independent prognostic factor in multivariate analysis. Similarly, in this study of RR-BCP-ALL, the age at transplantation was a significant prognostic factor. The outcome of haplo-transplantation was excellent in younger children under 10 years of age, with a 3-year OS rate of 100%, compared to a 3-year OS rate of 20% in children over 10 years of age (*p* < 0.002). The absence of TRM and recurrence with loss of mismatched HLA are possible reasons for the better prognosis in children under 10 years of age.

In this study, cumulative incidence of acute GVHD (grade II–IV) was 100% and that of grade III–IV acute GVHD was 70.1%. Of the 18 evaluable patients, grade II–IV acute GVHD developed in the skin of 16 patients, in the gut in 7 patients, and in the liver in 3 patients. Three out of 19 patients died from TRM; thus, the cumulative incidence of TRM was 15.8%. These three patients were over 10 years of age and died from EBV-lymphoproliferative disease, pulmonary hemorrhage due to invasive aspergillosis, or septic shock due to *Stenotrophomonas maltophilia*. The relatively low cumulative incidence of TRM (15.8%) despite the high incidence of GVHD suggests that in these extremely high-risk RR-BCP-ALL patients, some risk of acute GVHD should be tolerated and enhanced immunosuppression should be avoided to maintain the GVL effect. Nevertheless, due to the high risk of GVHD, our TCR-haplo-HSCT should be targeted at high-risk patients who are not eligible for conventional transplantation. Furthermore, since the 5-year leukemia free survival (LFS) of haplo-HSCT with T cell-depleted grafts for BCP-ALL patients who failed to achieve remission was 0% (23), the antitumor effect in TCR-haplo-SCT is clearly based on the allogeneic immune response by cytotoxic T-cells. In 2009, Vago et al. (24) reported that leukemic cells could escape the donor's antileukemic T cells through the loss of the mismatched HLA haplotype after haplo-SCT and that this mismatched HLA loss is caused by acquired uniparental disomy of chromosome 6p. This phenomenon is thought to cause tumor cells to evade attack by alloreactive donor T cells and cause relapse. Mismatched HLA loss after TCR-haplo-HSCT is a clonal evolution based on a high degree of genomic instability; at present, there is no effective way to predict or prevent it. We encountered a rare case (Patient No. 18) of BM relapse with the loss of maternal-derived mismatched HLA haplotype after TCR-haplo-HSCT using PBSCs from his father; after second TCR-haplo-HSCT using PBSCs from his mother, he relapsed again with the loss of paternal-derived mismatched HLA haplotype (17). Such leukemia cells with high genomic instability are likely to develop resistance to TCR-haplo-HSCT, making it difficult to maintain long-term remission. In fact, in this study, all three patients who relapsed owing to mismatched HLA loss died. While donor lymphocyte infusion (DLI) is considered for patients who had relapsed after TCR-haplo-HSCT, it could be dangerous if the presence of mismatched HLA loss is not confirmed. In addition, when a second TCR-haplo-HSCT is conducted, appropriate donor selection is needed after confirming the mismatched HLA loss in leukemic blasts.

The prognosis of patients with post-transplant relapse is dismal (25). A second transplantation for post-transplant relapse of ALL is associated with a high rate of relapse and poor long-term survival of 30% in 2-year OS (26). We conducted TCR-haplo-HSCT again in four out of eight relapsed patients after the first TCR-haplo-HSCT, and two of them are still alive. Of the two patients who died, one patient relapsed owing to loss of mismatched HLA and the other died of graft failure. The two surviving patients were both younger than 10 years of age, and there was no loss of mismatched HLA at the time of relapse after TCR-haplo-HSCT. Since these patients achieved remission with chemotherapy after the post-TCR-haplo-HSCT relapse, it could be suggested that clonal selection by strong allogeneic immunity filtered out and restored chemotherapy sensitivity. Thus, at the time of relapse after TCR-haplo-HSCT, a second TCR-haplo-HSCT may be considered if there is no loss of mismatched HLA and no organ damage in younger children. The limitation of this study is that it is a retrospective study with a small number of patients and was limited to a single center. A multicenter prospective clinical trial is currently underway to validate the efficacy of TCR-haplo-HSCT.

In conclusion, we suggest that our TCR-haplo-HSCT method has the potential to save the lives of RR-BCP-ALL patients with a very poor prognosis and no other treatment options. However, this study was conducted on a small number of patients at a single institution, and the results should be interpreted with caution. In addition, since the incidence of acute GVHD is extremely high, further improvement of the transplantation method needs to be considered. Prospective clinical studies are needed to further clarify the efficacy of this method.

## Data Availability Statement

The raw data supporting the conclusions of this article will be made available by the authors, without undue reservation.

## Ethics Statement

The studies involving human participants were reviewed and approved by Institutional Review Board, Fukushima Medical University. Written informed consent to participate in this study was provided by the participant's legal guardian/next of kin. Written informed consent was obtained from the minor's legal guardian/next of kin for the publication of any potentially identifiable images or data included in this article.

## Author Contributions

HS and AK conceived and designed the study and collected data from the medical records. HS was responsible for writing the manuscript. HO and KI contributed to this methodology. KM, SKo, and AK contributed to the review of this manuscript. All authors read and approved the final manuscript.

## Funding

This research was funded by the Department of Pediatric Oncology, Fukushima Medical University Hospital, who supported the open access publication cost.

## Conflict of Interest

The authors declare that the research was conducted in the absence of any commercial or financial relationships that could be construed as a potential conflict of interest.

## Publisher's Note

All claims expressed in this article are solely those of the authors and do not necessarily represent those of their affiliated organizations, or those of the publisher, the editors and the reviewers. Any product that may be evaluated in this article, or claim that may be made by its manufacturer, is not guaranteed or endorsed by the publisher.
